# Aerodynamic noise characteristics of a centrifugal fan in high-altitude environments

**DOI:** 10.1371/journal.pone.0296907

**Published:** 2024-01-18

**Authors:** Xue Liu, Jian Liu

**Affiliations:** 1 College of Safety Science and Engineering, Liaoning Technical University, Huludao, Liaoning, PR China; 2 Key Laboratory of Mine Thermo-motive Disaster and Prevention of Ministry of Education, Liaoning Technical University, Huludao, Liaoning, PR China; Wroclaw University of Science and Technology: Politechnika Wroclawska, POLAND

## Abstract

In high-altitude areas, the air is thin and the atmospheric pressure is low, which can affect the performance of centrifugal fans and aerodynamic noise. In this paper, steady and unsteady simulations of a centrifugal fan flow field are performed at altitudes of 0, 1000, 2000, 3000, 4000, and 5000 m, and the Ffowcs Williams-Hawkings equation is used to predict the aerodynamic noise of the fan. The results indicate that the tonal and broadband noise generated by the fan decrease with increasing altitude, and the A-weighted sound pressure level of each frequency band of the fan decreases when the air volume is held fixed. The maximum sound power level *L*_*wmax*_, sound pressure pulsation interval, and total noise sound pressure level *L*_p_ decrease linearly with increasing altitude. For every 1000 m increase in altitude, *L*_*wmax*_ and *L*_p_ decrease by 0.45 dB and 1.05 dB respectively. The fan noise characteristics, performance parameters, and human auditory perception are the main factors that affect the establishment of fan noise standards in high-altitude areas.

## Introduction

In recent years, China has accelerated the development and utilization of high-altitude areas in the west. However, in such areas, the air is thin, the atmospheric pressure is low, and the air contains less oxygen. Consequently, fluid machinery that uses air as a transmission medium often suffers from plateau hypoxia [1,2]. In places like factories, mines, and tunnels, centrifugal fans are frequently utilized for dust removal, ventilation, and equipment cooling; nevertheless, the noise issues they cause have sparked grave concerns [3,4]. For centrifugal fans to operate with high efficiency, low noise, and steady noise pollution management in high-altitude conditions, a detailed study of their aerodynamic noise characteristics is necessary [5,6].

Centrifugal fans generate two types of aerodynamic noise: discrete noise and broadband noise. Turbulent flow on the impeller and volute’s solid surfaces is thought to be the primary source of broadband noise. Tonal noise, or discrete noise, is generated when the fan volute and impeller output air flow interact [7,8]. The generation mechanism of fan aerodynamic noise has gathered considerable research attention, in addition to the development of noise numerical prediction methods and noise reduction design [9,10].

One of the primary techniques for researching centrifugal fan noise is experimental measurement. To explore the mechanism of aerodynamic noise, Velarde-Suarez et al. [11,12] recorded the pressure variation on the volute surface and sound pressure at the fan exhaust at various flow rates. They found that the unsteady force acting on the fan blade is another source of noise, and the discrete noise increases with increasing flow. After examining a fan’s internal flow, Sasaki et al. [13] hypothesized that the wake characteristics of a particular location in the scroll casing with a high relative flow velocity are what are causing the noise. Noise control technology has also gradually improved as the fan noise mechanism becomes better understood [14–16]. Qi et al. [17] used a variety of volute designs to examine the effects of inclined volute tongues, impeller blade tongue gaps, hub volute bearings, and their connection on the noise of centrifugal fans. The results of the experiments revealed that a well coupled modification can extend the operating range and improve fan performance while reducing fan noise. In addition to optimizing fan geometry for noise reduction purposes, some new materials (e.g., porous materials, micro-perforated plates, glass fibers) have also been shown to have good effects in absorbing fan noise [18,19]. Experiments performed by Xu et al. [20,21] showed that metal foam can effectively control fan noise.

Advances in computer science have propelled the application of CFD (computational fluid dynamics) and CAA (computational aeroacoustics) to predict centrifugal fan noise [22–24]. In the 1950s and 1960s, Powell developed the vortex sound theory and Lighthill proposed the acoustic analogy theory, both of which were important turning points in the development of aeroacoustics [25–27]. Building upon this foundation, the Proudman broadband noise model, Lowson discrete noise models, and FW-H (Ffowcs Williams-Hawkings) equations have been widely used to calculate fan aerodynamic noise [28–30]. Zhang et al. [31] used numerical analysis of a backward curved blade centrifugal fan to find the sound source position of the fan, compared the volute noise and blade noise intensity, and underlined the need of considering solid boundary conditions when evaluating the fan’s aerodynamic noise. Liu et al. [32] used the LES (Large Eddy Simulation) approach instead of the URANS (Unsteady Reynolds-Averaged Navier-Stokes) approach to accurately capture the flow separation when a fan rotates by simulating the unsteady fan flow process, and used the acoustic analog method to predict the fan noise. That research indicated that the primary source of sound for a fan is the dipoles distributed throughout the volute tongue surface. Using the indirect boundary element technique to account for the impacts of volute reflection and sound wave diffusing, Chen et al. [33] studied the noise of an industrial fan and increased the accuracy of their noise prediction. Darvish et al. [34] combined simulations and experiments and showed that a modification of the blade outlet angle can reduce a fan’s noise without compromising its aerodynamic performance.

In addition, with the rapid development of artificial intelligence technology, some intelligent methods have begun to be applied to the field of rotating machinery [35,36]. Kumar et al. [37] presented the development of a new convolutional neural network (NCNN) to efficiently identify bearing defects in rotating machinery from small samples. Vashishtha et al. [38] proposed a new optimisation method, amended gorilla troop optimization (AGTO), which enables the convolutional neural network (CNN) to adaptively extract features to identify worm gear defects.

Few studies have examined the aerodynamic noise of fans working in high-altitude situations, whereas the research mentioned above on the subject of centrifugal fan noise was primarily conducted in ordinary Earth-surface environmental circumstances. The internal flow state and aerodynamic features of centrifugal fans are influenced by changes in the physical properties of air that occur with altitude. However, the effects of these changes on the noise produced by a fan’s aerodynamic process are still not well understood. This work built a physical model of a centrifugal fan based on CFD and CAA techniques in order to answer these problems. Combining conventional and high-altitude environmental and climatic circumstances, the aerodynamic noise of a centrifugal fan was numerically simulated. The findings offer a theoretical framework for designing fans and creating noise regulations in high-altitude regions.

## Computational method

### Creation of physical models and meshes

**Fig 1:**
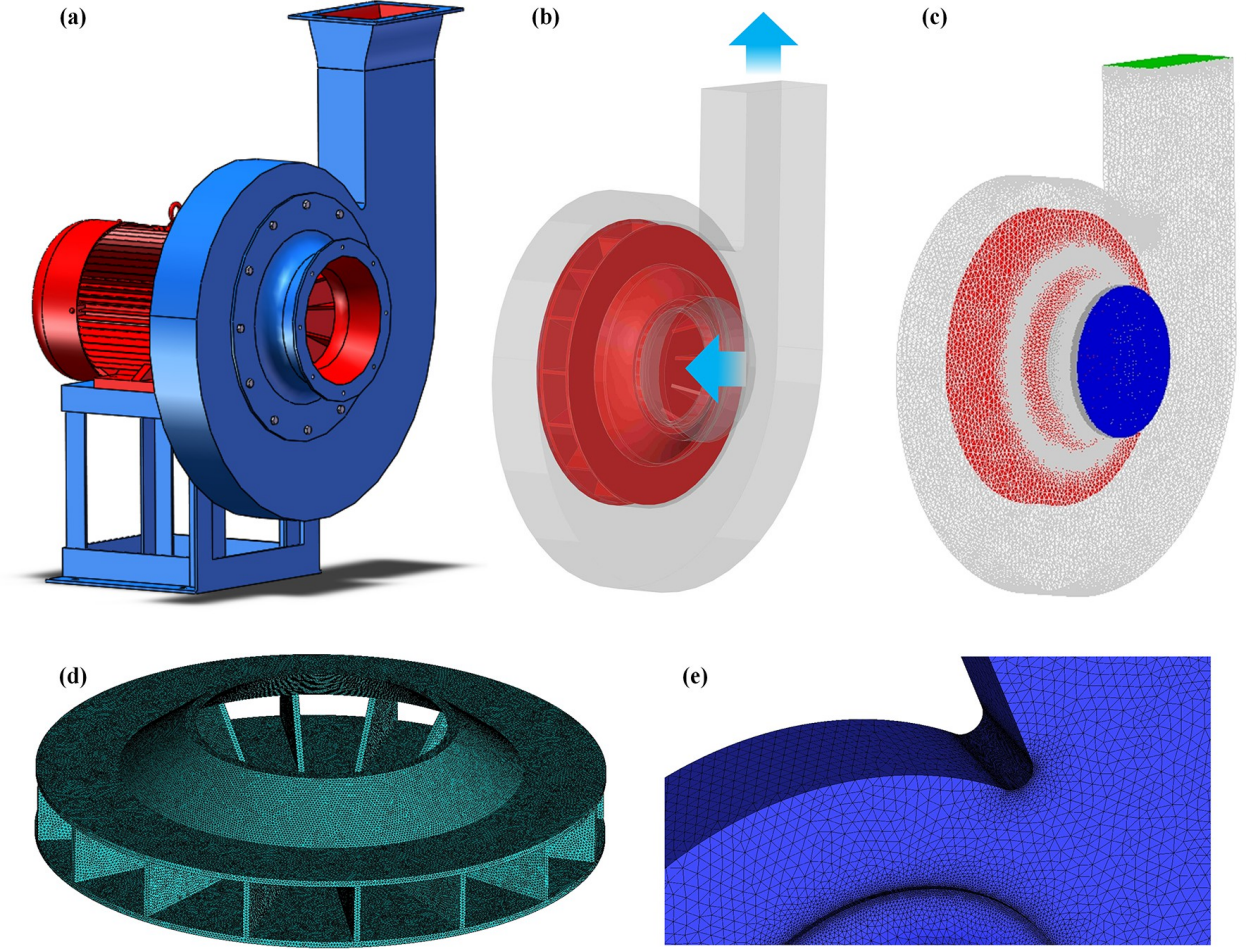
Fan model and grid division.

The research object in this paper was a 9-26-4A high-pressure centrifugal fan, and its essential structural parameters are provided in Table 1. As illustrated in Fig 1(A), a three-dimensional geometric model of the fan was created using SolidWorks software at a 1:1 ratio. The model was reasonably simplified for the simulation calculations, including the removal of the motor, pedestal, and other assembly parts while retaining the complete geometrical structure of the fan, to obtain a three-dimensional simulation model (Fig 1(B)). The fan model was imported into the ANSYS Workbench for meshing. The computation grid is created using unstructured tetrahedral pieces. A sparse grid is applied in the collector intake and volute outlet area, and a dense grid is applied in the impeller rotation area. The partition and local densification division methods are used. Fig 1(C) depicts the fan model’s overall grid division; Fig 1(D) and 1(E) show enlarged views of the localized grids at the impeller and volute tongue.

**Table 1:** Primary fan structural parameters.

Structural parameter	Numerical value
**Inlet diameter of collector**	112 mm
**Inlet blade angle**	90°
**Outlet blade angle**	90°
**Volute width**	90 mm
**Impeller outlet width**	50 mm
**Blade thickness**	5 mm
**Volute tongue radius**	10 mm
**Motor revolutions**	2900 rpm
**Number of blades**	16

### Grid independent verification

**Fig 2:**
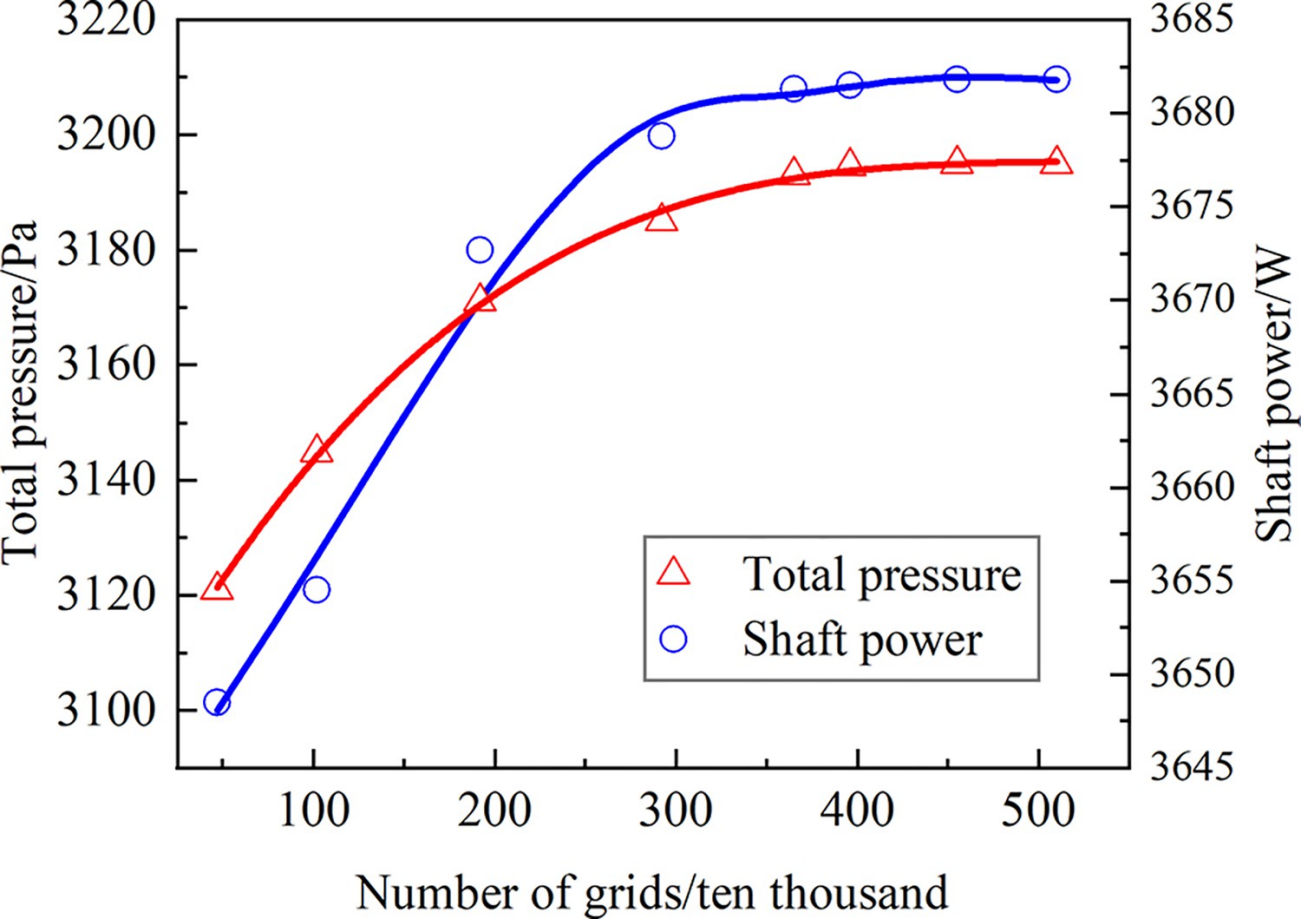
Grid independent verification.

The accuracy of numerical computation outputs is strongly influenced by grid size. Mesh division that is reasonable not only meets computation accuracy criteria but also reduces calculation costs. As seen in Fig 2, the relationship between the fan’s total pressure and shaft power with the number of model grids is depicted to confirm the grid’s independence when the air volume is 2190 m^3^/h under typical environmental circumstances. The image shows how the number of grids increases the fan’s total pressure and shaft power. When there are more grids than 4.52 million, the two don’t really change. A fan model with a grid number of 4.52 million is used for the following numerical computation to avoid the grid number interfering with the calculation results.

### Numerical computational method and boundary conditions

Two steps make up the simulation process: the stable and unsteady calculations. Fan noise and the internal flow field are solved with the ANSYS Fluent solution. Turbulence in the steady simulation is modeled using the realizable *k*-*ε* model [2,28,39]. The SIMPLE algorithm couples pressure and velocity, and solves the three-dimensional control equation in a second-order upwind manner to ensure calculation accuracy.

The velocity inlet is the definition of the fan collector inlet, and the inlet flow rates are successively set to 16.70, 17.88, 19.08, and 20.28 m/s at four operating points when the air volume is 2368, 2536, 2706, and 2877 m^3^/h, respectively. The definition of the pressure outlet is the volute outlet. The current collector and volute are set as a non-slip fixed wall surface, and the fan impeller is set as a rotating wall surface. The coupling between the rotating impeller and other fixed surfaces adopts a multiple reference frame model following the principle of relative motion [2,28,31,39].

After the computation is finished, the broadband noise model is presented in order to forecast the broadband noise of the fan numerically. The LES turbulence model is then initiated based on the Smagorinsky-Lilly subgrid model equation. The PISO algorithm performs pressure-velocity coupling. The pressure discrete format uses the PRESTO! format. A sliding mesh model is selected as the rotation domain, and a time step of 7 × 10^−5^ s is chosen. The pulsating pressure generated by the fan is monitored, and when the pulsation extreme value does not substantially fluctuate, the calculation is considered to have reached a stable state. Based on the three-dimensional unsteady computation, the FW-H model is then used to simulate the aerodynamic noise produced by the fan [2,28,31,39–41].

The ambient temperature in high-altitude areas is notably affected by seasons and weather, and there may be a large temperature difference within a given day, which is not easy to accurately estimate. However, the ambient air pressure decreases with increasing altitude. The relationship between the two is called the pressure altitude equation, which is described as follows according to high-altitude atmospheric statics theory and under the premise of isothermal conditions [2]:

$$P_{H}=P_{0}\exp(-\frac{g}{R_{g}T}\cdot H)$$ (1)

where *H* is the altitude, m/s; *g* is the acceleration of gravity, m/s^2^; *T* is atmospheric temperature, *K*; *R*_*g*_ is the gas constant; *P*_0_ is the atmospheric pressure in the plain environment, Pa; and *P*_*H*_ is the atmospheric pressure at altitude *H*, Pa.

**Fig 3:**
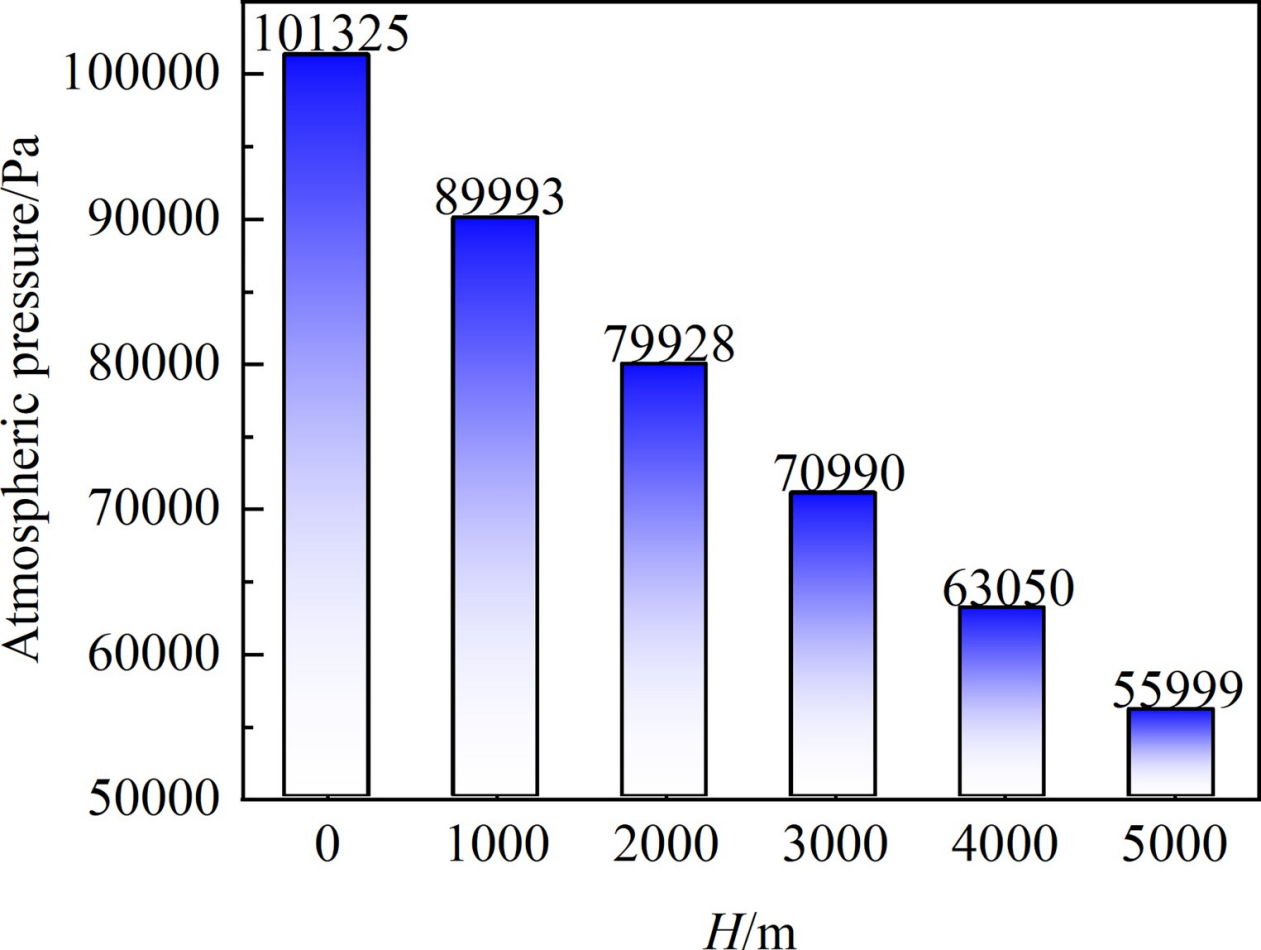
Relationship between altitude and atmospheric pressure.

**Fig 4:**
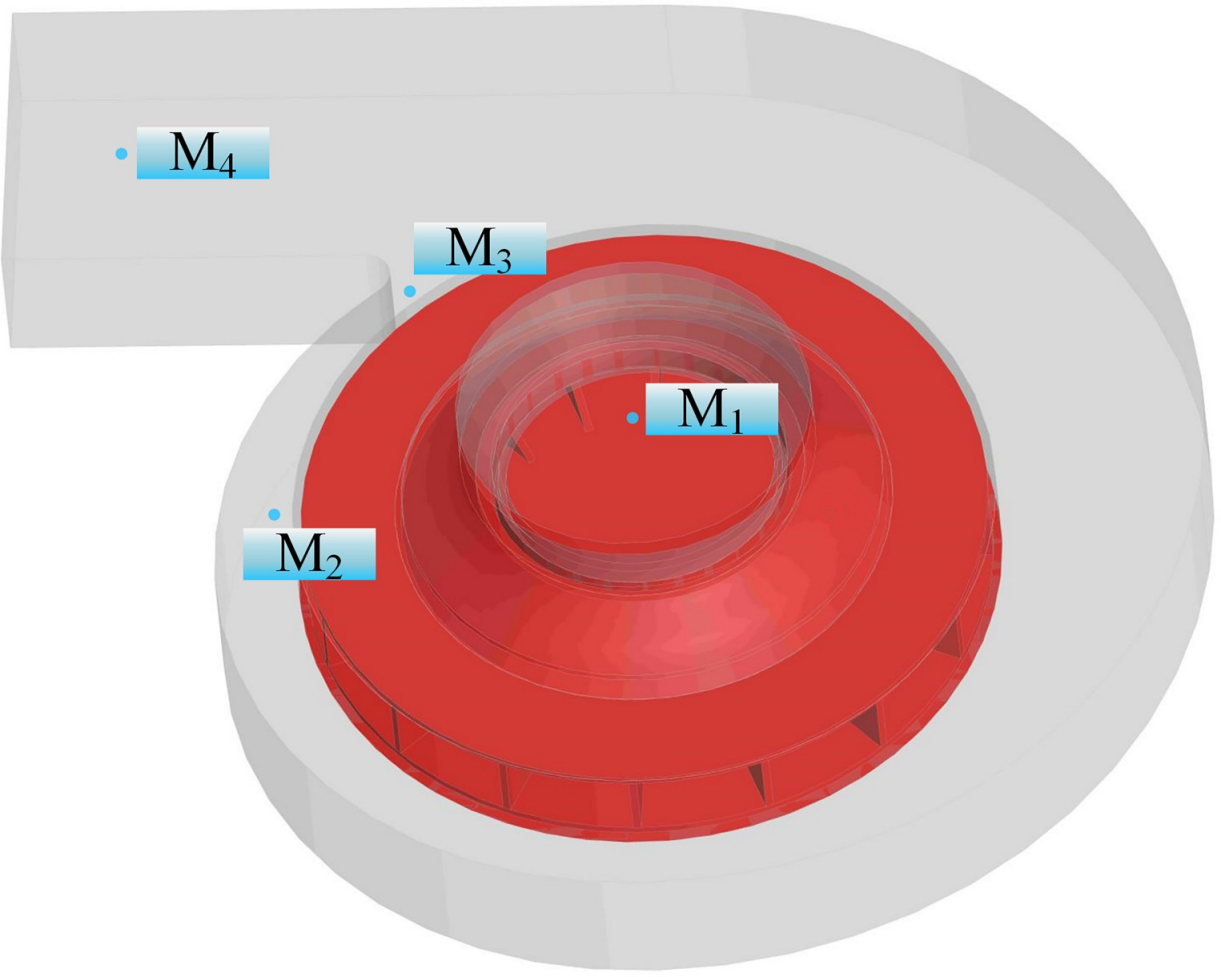
Arrangement of noise monitoring points.

Fig 3 illustrates the relationship between altitude and atmospheric pressure, with the environment temperature set at 15°C using Eq (1). At elevations of 0, 1000, 2000, 3000, 4000, and 5000 m, the centrifugal fan’s flow field and noise are computed and simulated. Noise monitoring points are set up in four typical positions near the fan inlet area (M_1_), impeller (M_2_), volute tongue (M_3_), and outlet area (M_4_), as shown in Fig 4.

### Experimental validation

**Fig 5:**
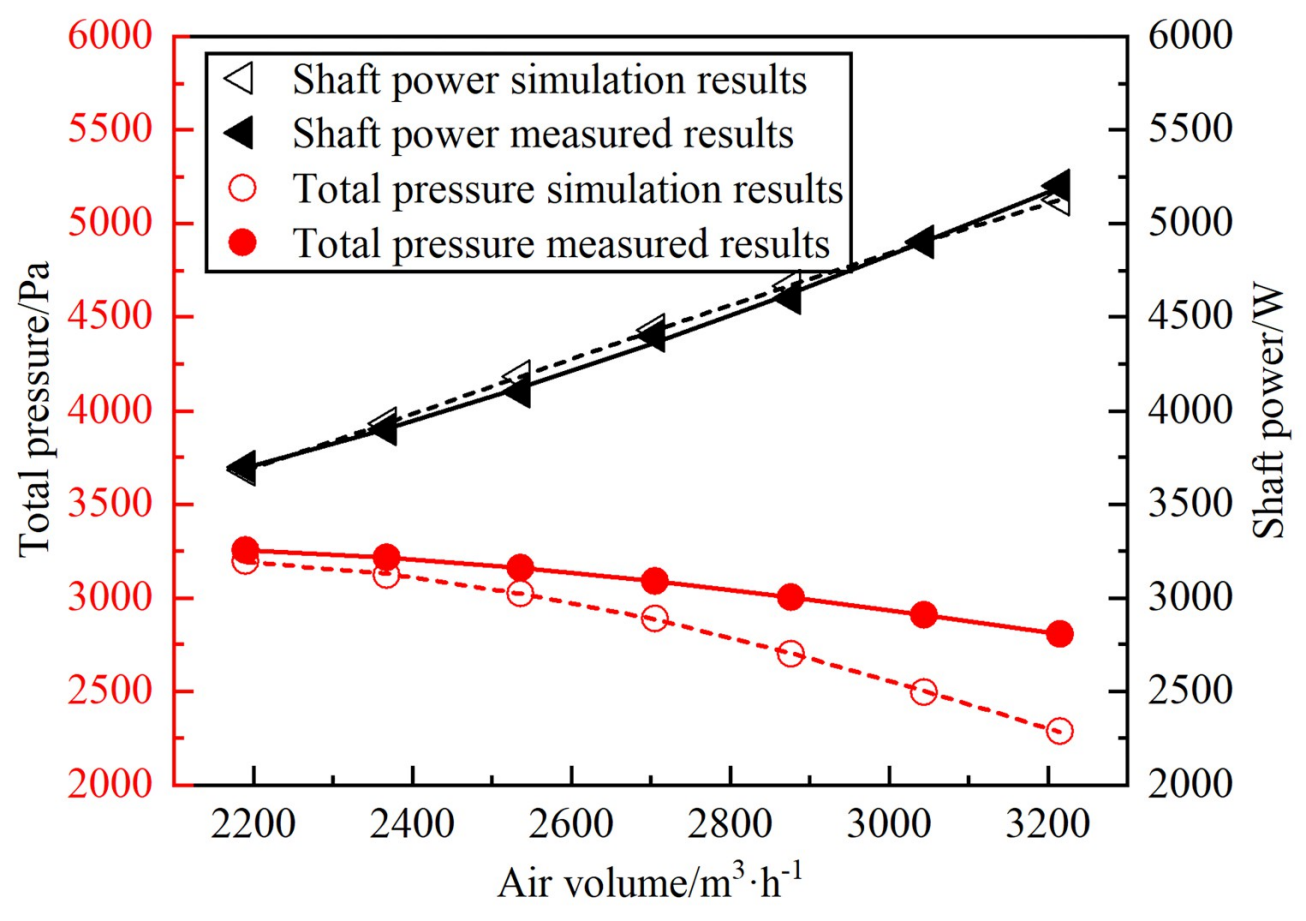
Experimental validation results.

In order to verify the reliability of the numerical method, the simulation results of the performance parameters of the 9-26-4A centrifugal fan under atmospheric pressure environment are compared and analyzed with the factory performance curve of the fan, as shown in Fig 5. From the figure, it can be seen that the shaft power and air volume is roughly linear relationship, the total pressure with the air volume is a non-linear trend of reduction. Under the same environmental conditions, the simulated fan shaft power and total pressure results are consistent with the overall trend of the actual results, and the numerical error is also within the acceptable range. Therefore, it is feasible to use numerical simulation to study the performance of the centrifugal fan.

## Results and discussion

### Acoustics prediction

**Fig 6:**
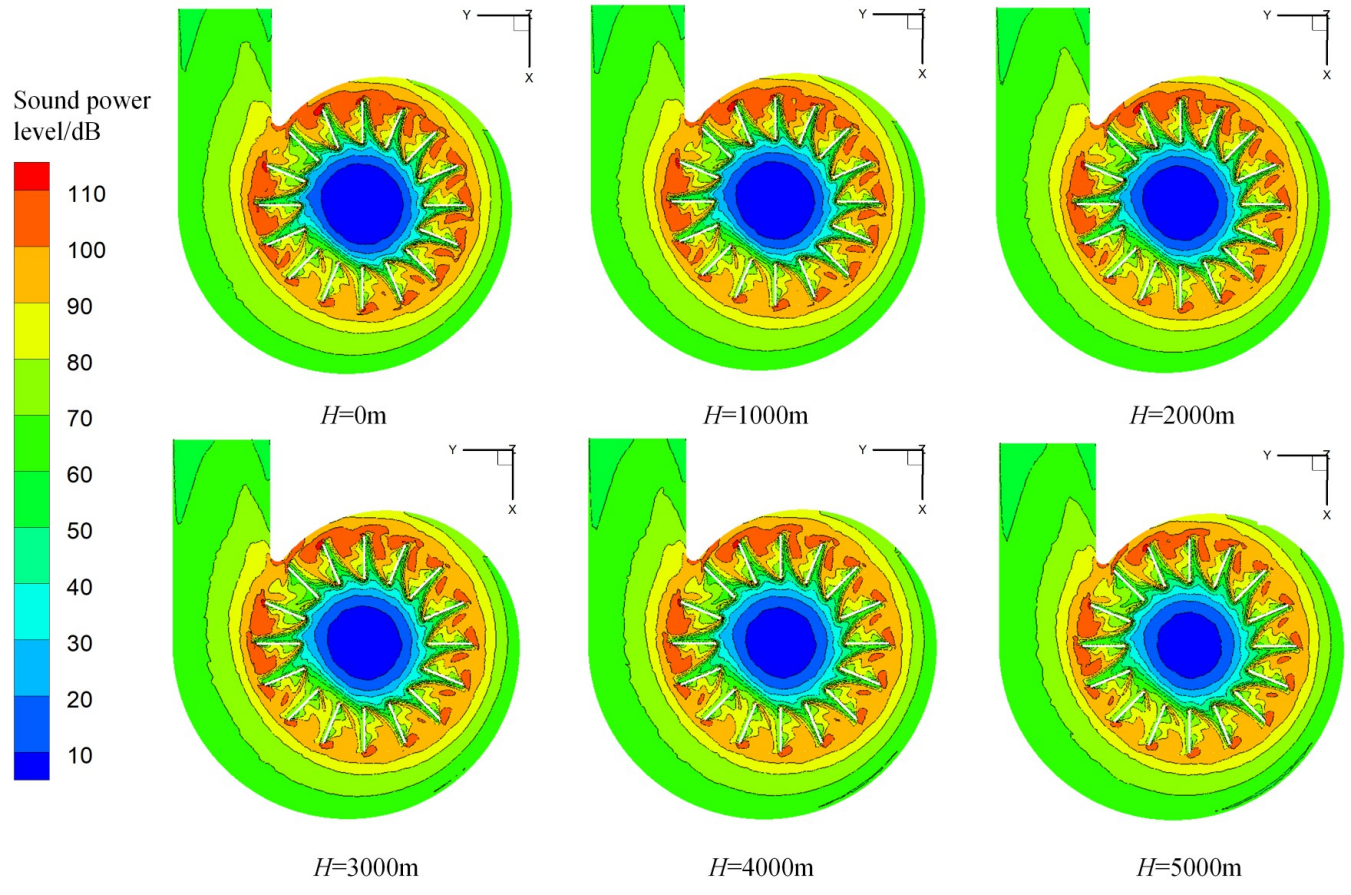
Cloud graphs showing the distribution of sound power levels at various altitudes in the fan’s central section.

Sound power is an important parameter to measure a sound source’s ability to radiate sound. Cloud graphs showing the sound power level distribution in the fan’s central section at several altitudes for a fan air volume of 2706 m^3^/h are displayed in Fig 6.

The shape of the sound power level distribution cloud diagram inside the fan is similar over the full range of investigated altitudes (Fig 6), whereas the sound power is notably non-uniform owing to the asymmetric structure of the volute. Due to the separation of the rotating impeller’s wake, there is more sound power in the tip region of a single blade and a higher sound power level in the vicinity of the suction surface than in the vicinity of the pressure surface. The maximum sound power level occurs around the tip of the second blade that starts counterclockwise along the volute tongue under all of the investigated altitude conditions. The sound power level gradually decreases as the blade moves away from the volute tongue area. Under the same working conditions and air volume conditions, the sound power level of various parts inside the fan generally decreases with increasing altitude, which indicates a weakening trend of the radiated noise intensity of the fan’s sound source surface.

**Fig 7:**
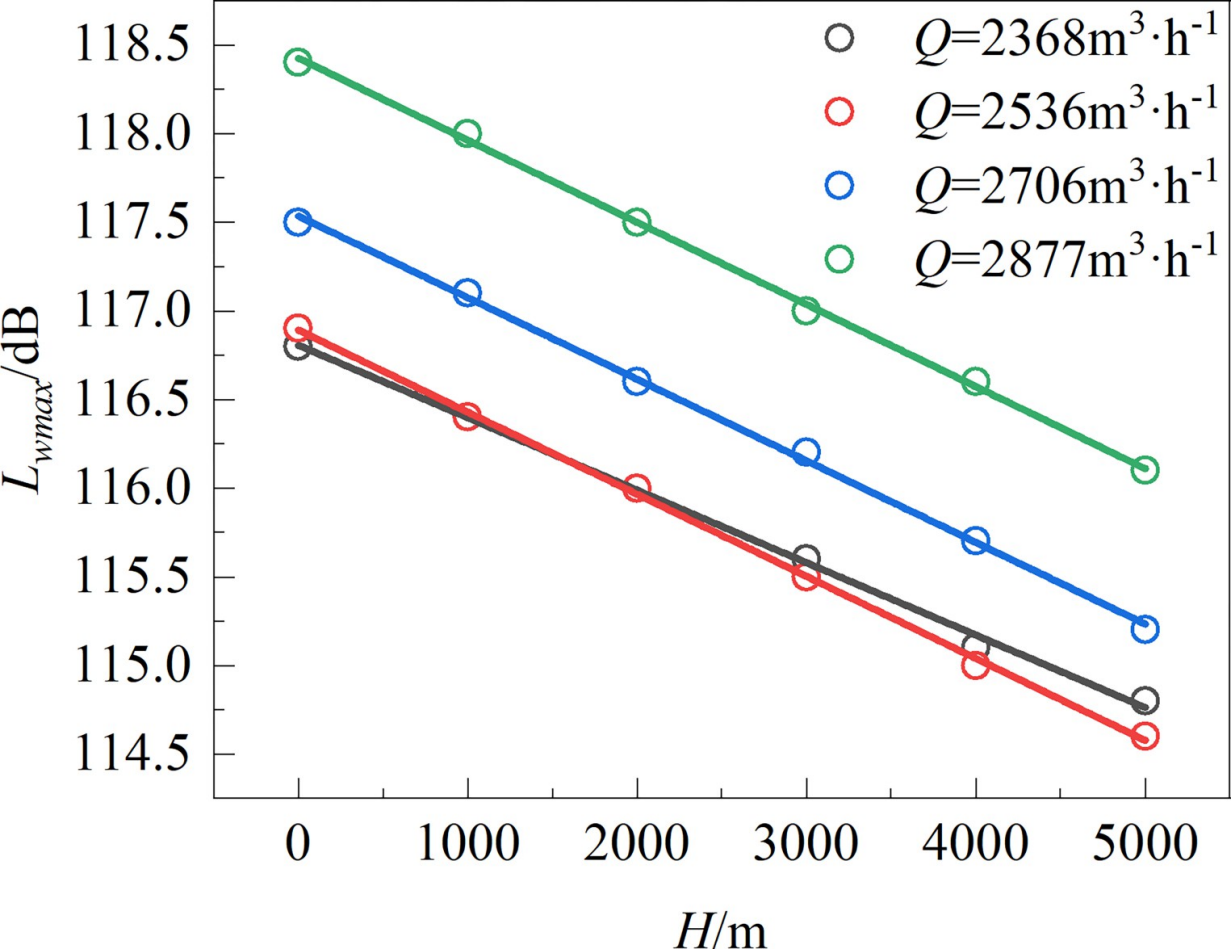
Relationship between altitude and maximum sound power level.


Fig 7 quantitatively analyzes the effect of height on the broadband noise of the fan by plotting the relationship between altitude *H* and maximum sound power level *L*_*wmax*_. The *L*_*wmax*_ values of working conditions 1 and 2 are relatively similar at a given altitude and increases with increasing air volume. The fitting equation between the maximum sound power level *L*_*wmax*_ and the altitude *H* is shown in Formula (2). The results indicate that *L*_*wmax*_ decreases linearly with increasing *H*. When the air volume is held constant, the fan’s *L*_*wmax*_ value decreases by approximately 0.45 dB per 1000 m of altitude increase.

$$\left\{ \begin{array}{l} L_{wmax}=(4.09\times 10^{-4})H+116.80\;Q=2368m^{3}\cdot h^{-1}\\ L_{wmax}=(4.63\times 10^{-4})H+116.89\;Q=2536m^{3}\cdot h^{-1}\\ L_{wmax}=(4.60\times 10^{-4})H+117.53\;Q=2706m^{3}\cdot h^{-1}\\ L_{wmax}=(4.63\times 10^{-4})H+118.42\;Q=2877m^{3}\cdot h^{-1} \end{array}\right.$$ (2)

### Time domain characteristics of noise signal

**Fig 8:**
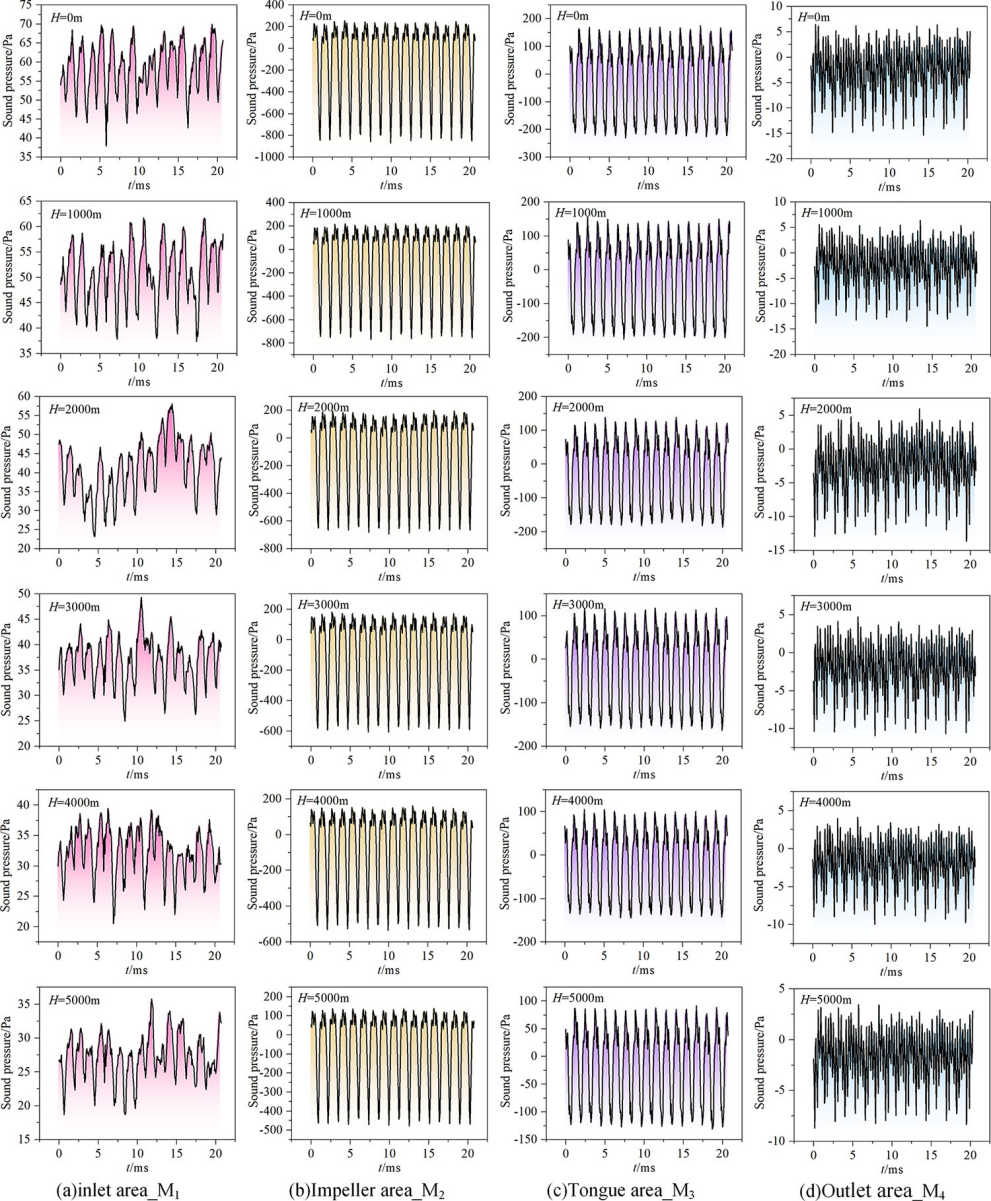
The relationship between sound pressure and altitude.

To investigate the fluctuation of the sound pressure signal of the aerodynamic noise produced by the fan in a high-altitude environment, working condition 3 is used as an example. At various altitudes, the sound pressure signal’s temporal domain information is retrieved. The relationship between the sound pressure and time in a specific rotation period is depicted in Fig 8.

The monitoring stations situated in the impeller area and volute tongue display sound pressure readings that display a consistent periodic pulsation pattern, with distinct peaks of comparable sizes. The number of peak points-16-is equivalent to the model fan’s blade count. This is because the blades are evenly distributed along the circumference, and the number of rotations remains constant. The excitation effect generated by the blower blades against the surrounding air is therefore presented in periodic form. The sound pressure signal at monitoring point M_1_ in the entrance area also shows a peak point with a pulsation amplitude that is consistent with the number of blades; however, differing from M_2_ and M_3_, the magnitude of each amplitude differs substantially and exhibits characteristics of similar periodic pulsations. For the same kind of periodic pulsation, the sound pressure time domain signal at M_4_ in the outlet area becomes denser and the peak point is no longer apparent. From a numerical viewpoint, the sound pressure pulsation values range from large to small in the impeller area, volute tongue area, inlet area, and outlet area. The unstable aerodynamic action between the blade and volute tongue affects monitoring point M_2_ in the impeller area, leading to a significant absolute value of the sound pressure pulsation. While the sound pressure at M_1_ continuously remains positive, it alternatively pulses between positive and negative at monitoring stations M_2_, M_3_, and M_4_.

**Fig 9:**
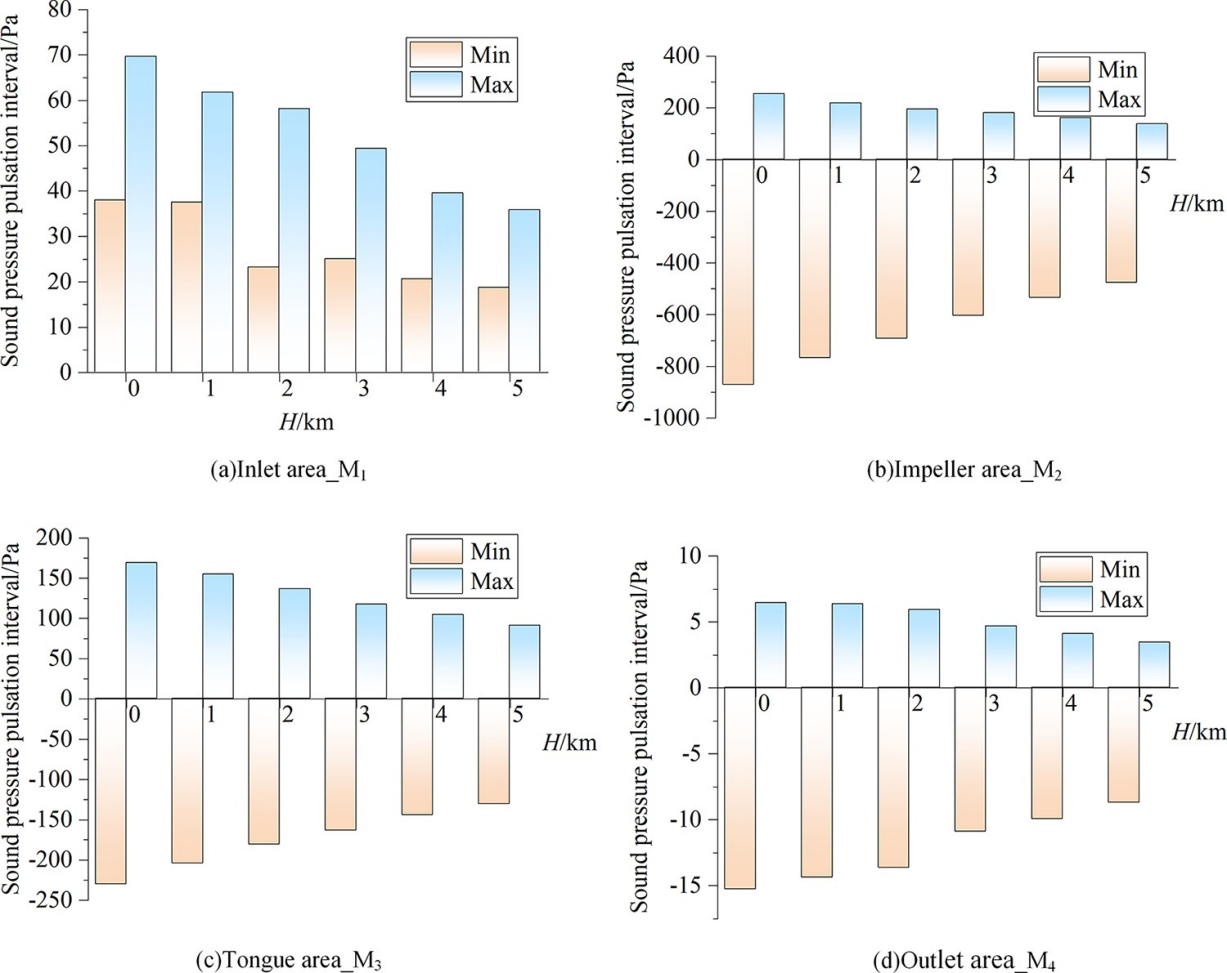
Sound pressure pulsation intervals of the fan under different altitude conditions.

Aside from a slight change of the sound pressure signal in the entrance area, the shape of the sound pressure signal at the other three measurement points remains essentially unchanged with increasing altitude, but the sound pressure pulsation amplitude gradually decreases (i.e., the noise signal is weakened). Fig 9 illustrates the quantitative link between altitude and the noise sound pressure pulsation interval. It is discovered that as altitude increases, both the pulsation interval range and the absolute value of the sound pressure pulsation extreme value eventually decrease. The calculations indicate that under a given set of working conditions and air volume conditions, the sound pressure pulsation interval at *H* = 5000 m is reduced by roughly 55% compared with the standard environment, which means that the pulsating sound pressure decreases by approximately 11% per 1000 m of altitude increase.

### Frequency domain characteristics of noise signal

**Fig 10:**
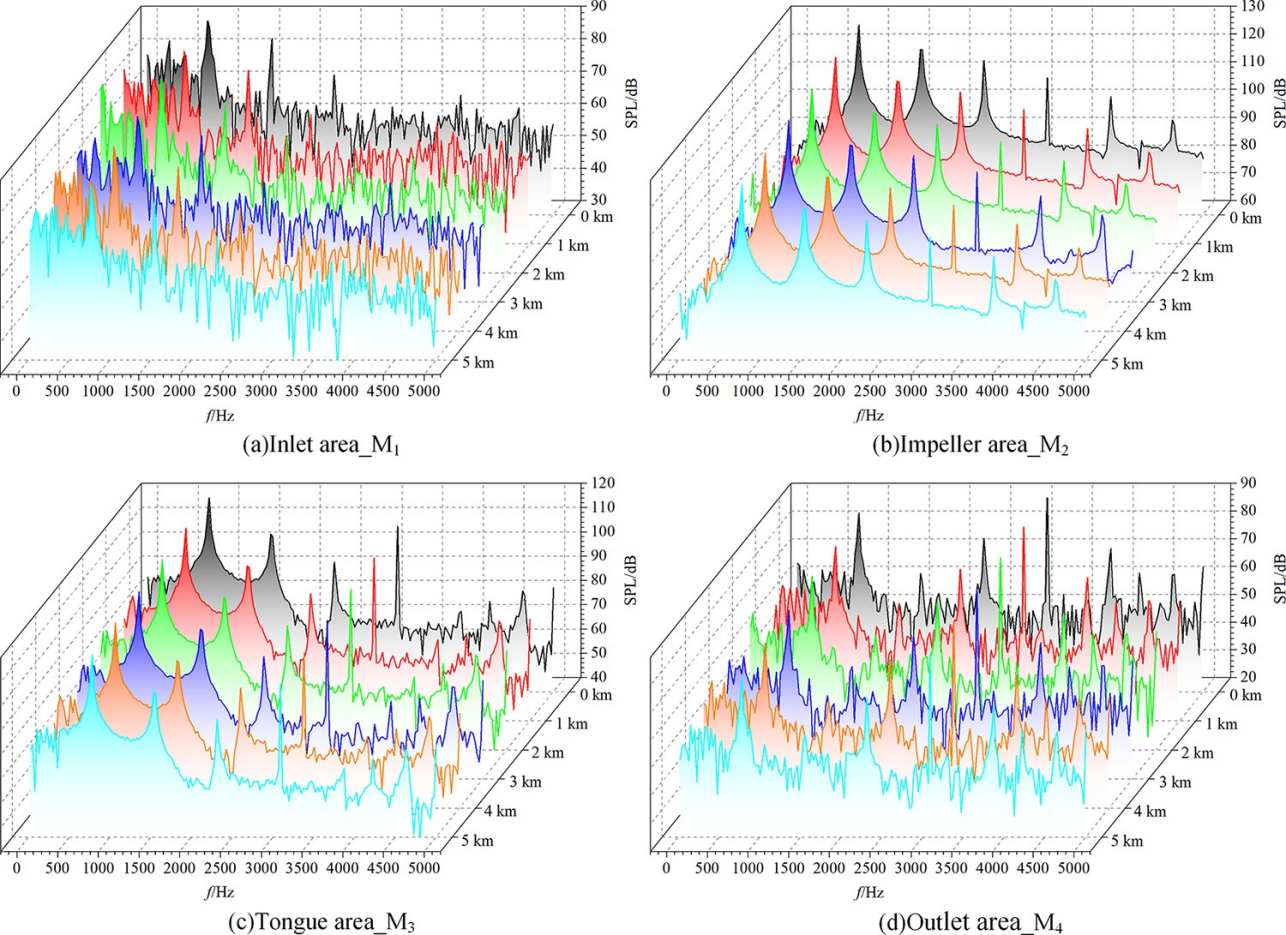
Fan noise spectrum.

The sound pressure time domain data is subjected to FFT (Fast Fourier Transform) in order to get the fan noise spectrum curve (Fig 10) and analyze the noise distribution features in the frequency domain at the fan’s measurement locations under varied altitude conditions. The form of the noise’s frequency spectrum at the measurement sites generally varies significantly, reflecting the variations in the kinds of noise present in the various fan zones. The noise signal in the impeller area shows a relatively regular harmonic form, which indicates that this area mainly involves discrete noise. The frequency formula of discrete noise is given as:

$$f=\frac{nZ}{60}i$$ (3)

where *n* is the number of fan revolutions (rpm), *Z* is the number of fan blades, and *i* represents the harmonic sequence number.

Based on calculations, the centrifugal fan’s fundamental frequency is 773.3 Hz, which matches the noise spectrum curve. Within the frequency range of 0–5000 Hz, the fundamental frequency has the highest noise sound pressure intensity, which progressively diminishes as the number of harmonics increases. Discrete noise characteristics are notable when the frequency of the volute tongue region is in the range of 0–2500 Hz, and discrete and broadband noise occur simultaneously with increasing frequency. The inlet and outlet zones consistently maintain a state of inter-doping discrete and broadband noise within the calculated frequency.

The high-altitude environment’s noise spectrum form does not considerably differ from the conventional environment’s (Fig 10), but each frequency band’s sound pressure level value is significantly lower, suggesting a reduction in both discrete and broadband noise. According to the fan sound principle, when a fluid flows through the blade, a boundary layer forms on the surface that can easily thicken, particularly on the suction surface. The separation of the boundary layer therefore produces several vortices. The wake area has significantly lower airflow pressure and velocity than the main airflow area, and the resulting lateral airflow movement promotes the formation of wake vortices. The unevenness of the airflow in the blade exit area is therefore significant when the working wheel rotates. This uneven airflow periodically acts on the surrounding medium, which generates various types of vortices and causes pressure pulsation and noise [24].

**Fig 11:**
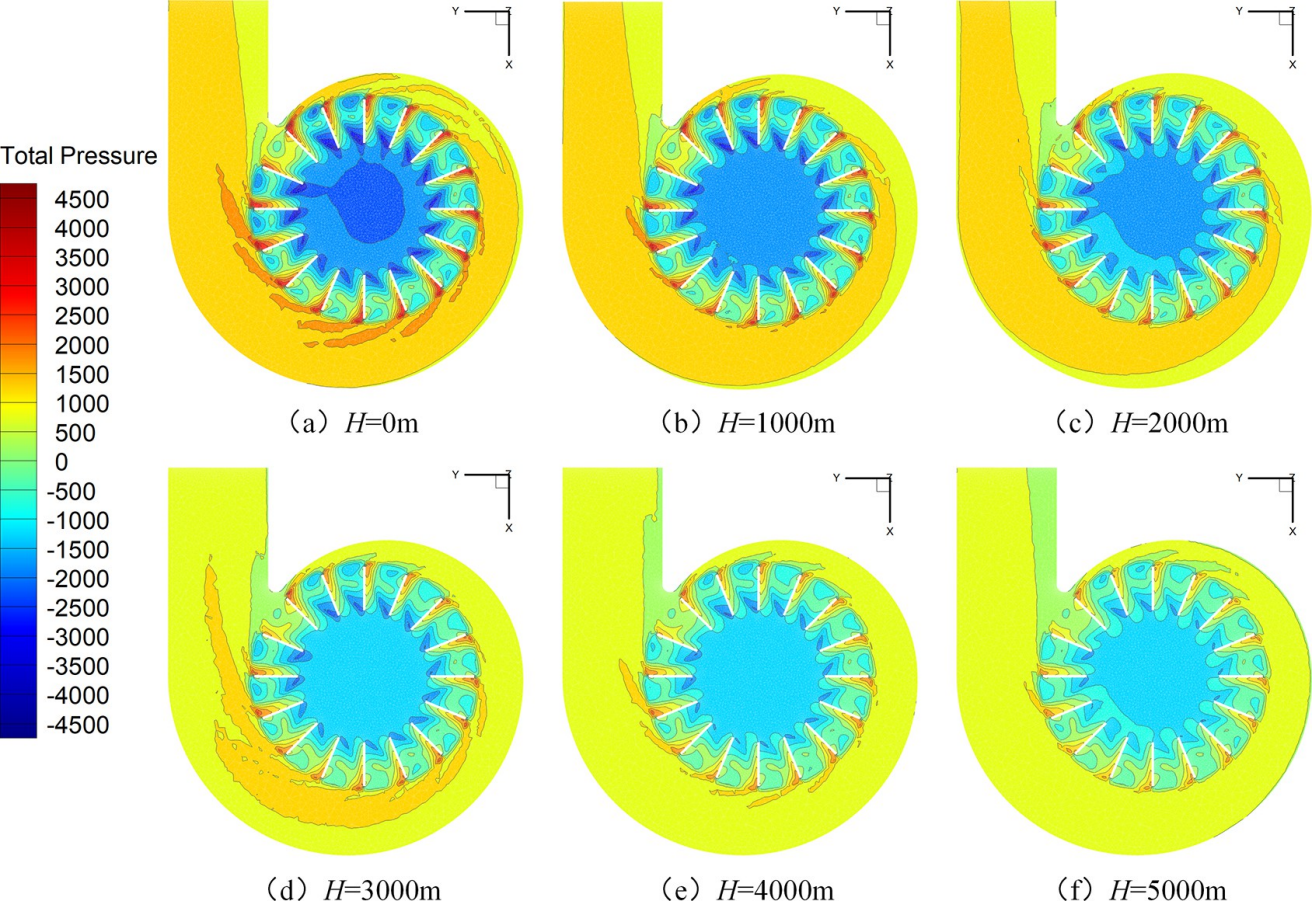
Total pressure cloud diagram of the fan.

The total pressure distribution of the fan’s middle section under various situations can be examined in order to investigate the causes behind the decrease in fan noise at high altitude (Fig 11). Although the figures are significantly different, the total pressure distribution within the fan is similar. The atmosphere gets thinner and the activity of gas molecules reduces as one ascends higher into the sky. The influence of the blade rotation on the surrounding air is lessened for a given number of fan rotations, which lowers the internal flow field pressure overall. Additionally, Fig 11 demonstrates how a lateral pressure gradient is formed by positive and negative pressure zones that form close to the fan blade’s pressure and suction surfaces. As a result, air moves from the suction surface to the pressure surface. As altitude increases, the fan’s vortex diminishes because of a lessened lateral pressure gradient, which lowers noise levels.

In order to objectively assess the impact of altitude, the total sound pressure level of the noise can be computed using the following formula:

$$L_{p}=10\times \mathrm{lg}(\sum_{i=1}^{n}10^{0.1\times L_{\mathrm{pn}}})$$ (4)

where *L*_p_ is the total sound pressure level of the noise (dB) and *L*_pn_ is the sound pressure level corresponding to each frequency in the spectrum (dB).

**Fig 12:**
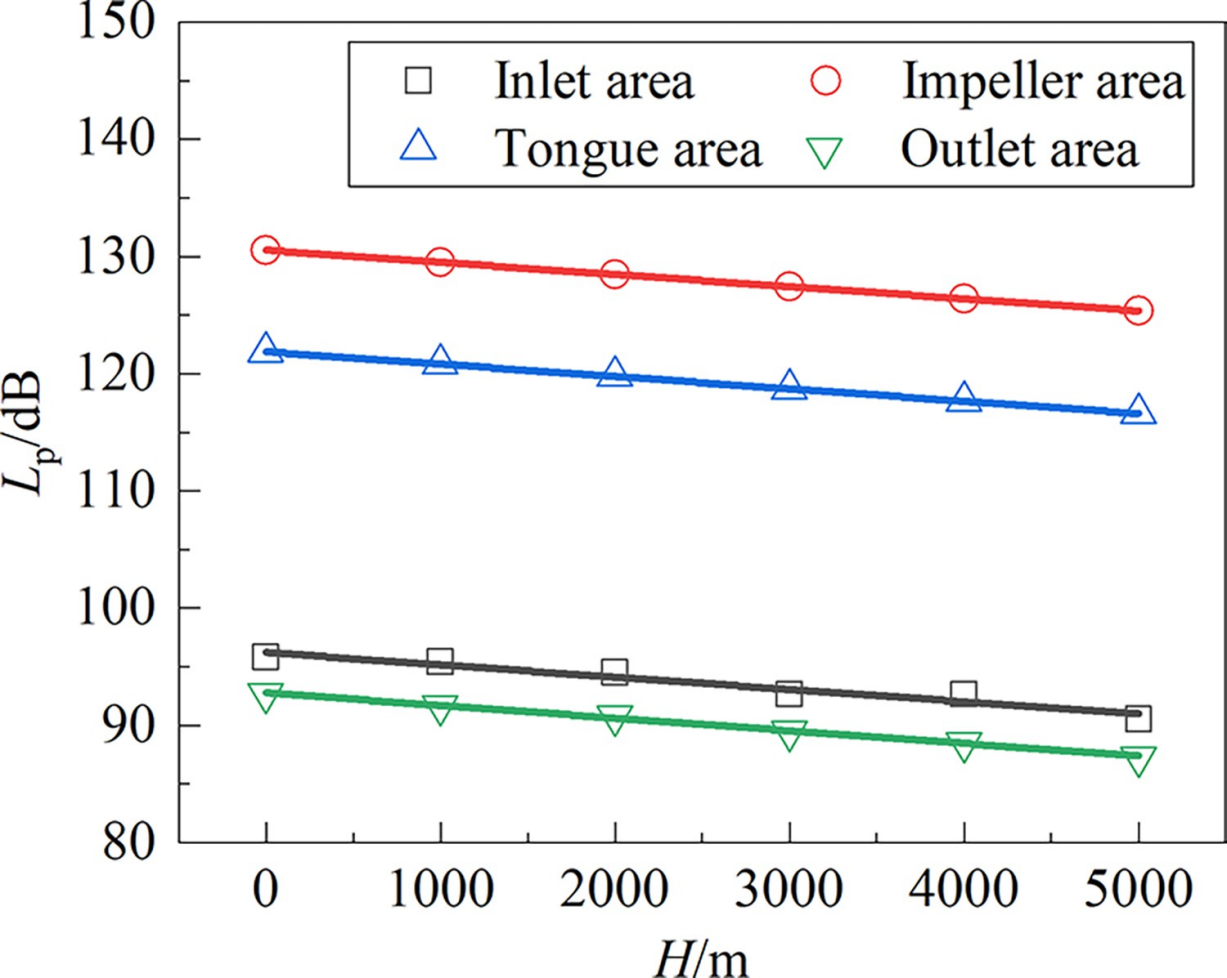
Relationship between the *L*_P_ and altitude.

Fig 12 displays the relationship curve between the altitude and the total sound pressure level of the fan noise. With increasing altitude, the overall sound pressure level of the noise typically falls linearly, with each area seeing a similar declining tendency. The equation of the fitted relationship between *L*_p_ and *H* is shown in Formula (5). Every 1000 meters of altitude gain is observed to result in a fall of about 1.05 dB in the overall sound pressure level.


$$\left\{ \begin{array}{l} L_{p}=-0.00105H+96.22\;Inlet\;area\;Inlet\;area\\ L_{p}=-0.00103H+130.52\;Impeller\;area\\ L_{p}=-0.00103H+130.52\;Tongue\;area\\ L_{p}=-0.00108H+92.79\;Outlet\;area \end{array}\right.$$ (5)

**Fig 13:**
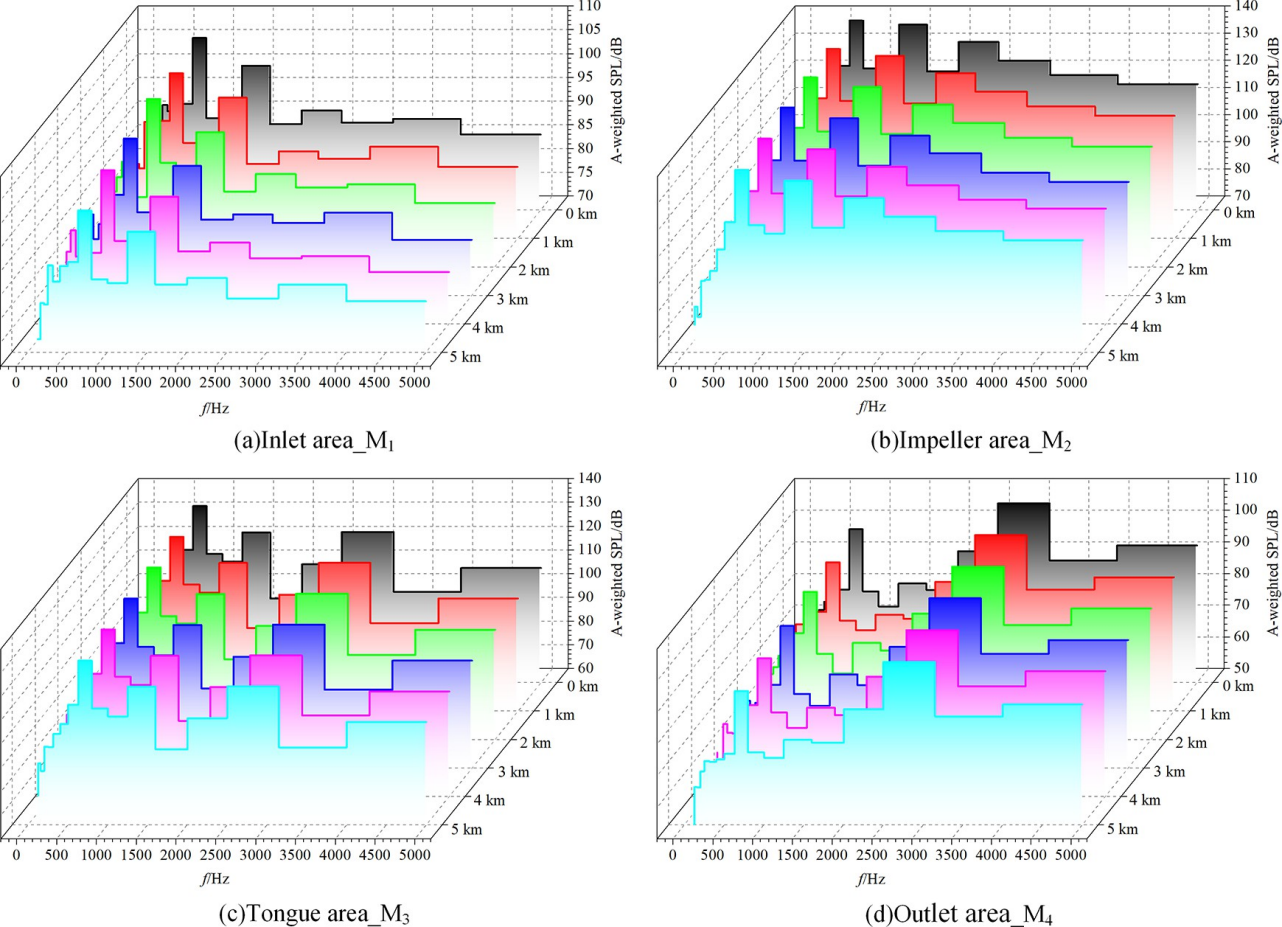
A-weighted 1/3 octave frequency spectra.

One of the primary benchmarks for evaluating noise is the A-weighted sound pressure level, which is a good representation of people’s natural perceptions of loudness. **Fig 13** shows the A-weighted 1/3 octave frequency spectra of each area under different altitude conditions. The inlet zone and impeller zone are mainly low-frequency noise, whereas the volute tongue zone and outlet zone have peaks in the middle, high, and low frequencies. Each frequency’s A-weighted sound pressure level typically drops as altitude rises. This shows that the noise level of the fan that is audible to humans in a high-altitude environment is lower than in a conventional environment for a given number of fan rotations and air volume.

With increasing altitude, the atmosphere becomes thinner and the number of gas molecules per unit volume decreases. The blowing effect of the rotating blades on the surrounding air at the same fan speed is reduced and the overall pressure pulsation is reduced, which ultimately leads to a reduction in fan noise.

As per the People’s Republic of China’s Machinery Industry Standard JB/T 8690–2014 "Ventilator Noise Limits" (also known as fan noise standards), a centrifugal fan with radial blades shall have a ratio of the A-weighted sound pressure level that is less than or equal to 22 dB. At a test operating point, the ventilator noise ratio of the A-weighted sound pressure level is as follows:

$$L_{\mathrm{SA}}=L_{A}-10\mathrm{lg}(Qp^{2})+19.8$$ (6)

where *L*_A_ is the A-weighted sound pressure level at the air inlet (or outlet) (dB), *Q* is the flow rate at the test operating point (m^3^/min), *p* is the pressure at the test operating point (Pa), and *L*_SA_ is the ratio of the A-weighted sound pressure level at the air inlet (or outlet) of the fan (dB).

The *L*_SA_ values of the centrifugal fan at altitudes of 0m, 1000m, 2000m, 3000m, 4000m and 5000m are 18.6dB, 18.3dB, 18.1dB, 17.8dB, 17.4dB and 17.0dB, respectively, as calculated in Formula (6). It can be seen that the noise of the 9-26-4A centrifugal fan meets the requirement of "Ventilator Noise Limits". In addition, the *L*_SA_ value of the fan shows a linear decreasing trend with increasing altitude, and the fitting equation of the relationship between the two is:

$$L_{\mathrm{SA}}=(-3.14\times 10^{-4})H+18.65$$ (7)

Atmospheric pressure decreases with altitude, and different fan performance parameters will impose large changes (e.g., output wind pressure, power) for a given number of revolutions and air volume of the fan. The atmospheric oxygen content in high-altitude areas also decreases and people can experience altitude sickness (e.g., dizziness, anxiety), accompanied by a weakening of the human ear’s ability to withstand noise. The geographical and climatic features of an area should therefore be fully integrated in the formulation of fan noise standards in high-altitude areas. It is recommended to consider the following three factors: 1) fan noise characteristics in high-altitude environments; 2) fan performance parameters in high-altitude environments; and 3) the impact of altitude on human hearing.

### Conclusion

For a given air volume, the shape of the sound power distribution cloud diagram inside a centrifugal fan does not particularly change with increasing altitude, but the values decrease. The maximum sound power level *L*_*wmax*_ decreases roughly linearly by approximately 0.45 dB per 1000 m of altitude increase.

The sound pressure signal form in the inlet zone changes slightly with increasing altitude. The sound pressure signal forms in the impeller zone, volute tongue zone, and outlet zone remain basically unchanged, but the amplitude of the sound pressure pulsation in each zone decreases. The range of the pulsation interval is reduced and the noise signal is weakened. The sound pressure pulsation interval at *H* = 5000 m is reduced by roughly 55% compared with the standard environment, which means that the pulsating sound pressure decreases by approximately 11% per 1000 m of altitude increase.

The atmospheric pressure is lower in high-altitude environments, and the discrete and broadband noise generated by the fan are simultaneously reduced. The total noise sound pressure level *L*_p_ decreases linearly with increasing altitude along a generally similar decreasing trend in each area. The *L*_p_ of the fan decreases by approximately 1.05 dB per 1000 m of altitude increase. The A-weighted sound pressure level *L*_A_ of each frequency of the fan is less in high-altitude environments than in a standard environment.

Fan noise characteristics, performance parameters, and human auditory perception are the main factors that affect the establishment of fan noise standards in high-altitude areas.

## Supporting information

S1 Dataset (XLSX)
